# Determinants of Honey and Other Bee Products Use for Culinary, Cosmetic, and Medical Purposes

**DOI:** 10.3390/nu15030737

**Published:** 2023-02-01

**Authors:** Iwona Kowalczuk, Jerzy Gębski, Dagmara Stangierska, Agata Szymańska

**Affiliations:** 1 Department of Food Market and Consumer Research, Institute of Human Nutrition Sciences, Warsaw University of Life Sciences-SGGW, Nowoursynowska 159C, 02-776 Warsaw, Poland; 2 Department of Pomology and Horticulture Economics, Institute of Horticulture Sciences, Warsaw University of Life Sciences-SGGW, Nowoursynowska 166, 02-787 Warsaw, Poland

**Keywords:** bee products, consumers behaviour, Poland, nutritional knowledge, health self-assessment

## Abstract

Bee products have been used for centuries for culinary, medicinal, and cosmetic purposes, and their properties are still a subject of research, which provide new arguments in favour of their use. The research aimed to determine the current state of use of bee products by Polish consumers and determine the ways and conditions of their use, with particular reference to the level of nutritional knowledge and health status. The survey was conducted using the CAWI (Computer-Assisted Web Interview) method on 487 respondents. It was found that honey is used mainly for culinary purposes and, to a lesser extent, for medicinal and cosmetic purposes. Other bee products are much less commonly used than honey—mainly beeswax and royal jelly for cosmetic purposes and propolis and bee pollen for medicinal purposes. Segments distinguished by the frequency of use of honey for particular purposes were differentiated by gender, age, income level, use of other bee products, and motivation to use them. Their differences were also found in terms of the level of nutritional knowledge and self-assessed health status—the highest ratings in both categories were indicated by representatives of the Honey users’ segment, which consisted of people who use honey most frequently for cooking, cosmetic and medicinal purposes. Regression analysis additionally showed that higher levels of nutritional knowledge and better health status were associated with the use of honey to treat gastrointestinal ailments and with the use of propolis for medicinal purposes.

## 1. Introduction

The properties of bee products were already known in ancient Egypt, Greece, and China. Their benefits are mentioned in many religious texts, including the Vedas, the Bible, and the Koran [1]. Historical sources indicate that in the past, honey was primarily a sweetener and a symbol of prosperity [2]. Its medicinal properties were also used extensively [3]. Over time, honey and other bee products have been replaced by functional foods and pharmaceutical preparations; however, there is a renaissance of interest in these products in an era of reversion to traditional nutrition and a growing interest in natural medicine [4]. This is undoubtedly due to the numerous scientific studies confirming the beneficial effects of bee products on the human body [5,6].

The most widely used bee product is undoubtedly honey. It is produced by *Apis mellifera* bees from the nectar of plants, the secretions of living parts of plants, or the secretions of insects sucking the living parts of plants [7]. Honey contains approximately 80–95% sugar [8,9], which determines its culinary use as a sugar substitute in beverages, cakes, desserts, dishes, and preparations. The alluring taste of honey also makes it ideal for direct consumption, as a spread on sandwiches or as a side dish. Valuable nutrients are present in honey, including amino acids of physiological importance [10] and bioactive components such as vitamins, phenols, flavonoids, fatty acids, and organic acids [11,12], determining its nutritional value and health-promoting effects. Honey is used in the prevention and treatment of conditions such as cardiovascular diseases, cancer, and diabetes [13]; skin diseases and hard-to-heal wounds [14,15]; or oral diseases [16]. The immunomodulatory, anticancer, anti-hypertensive, anti-allergic, and prebiotic effects of honey [13], its beneficial effects on patients with endocrine disorders [17], and its positive effects on fertility [18] have also been proven. Honey is also used in cosmetics to regenerate and moisturise the skin [19]. The fruit acids in honey determine its exfoliating effect. The presence of flavonoids prevents skin irritation during exposure to sunlight [20]. Adding honey to cosmetic preparations improves skin tone, colour, and elasticity [19].

Bee pollen is pollen collected by bees to replenish their food supply. The composition of the pollen, including the content of fatty acids, nucleic acids, nucleosides, phenolic compounds, vitamins, and bioelements, determines its therapeutic properties [21]. Previous studies have found that bee pollen can be used in the treatment of prostatitis, acute and chronic hepatitis; it regulates intestinal function, inhibits histamine reactions that cause allergy symptoms, and helps maintain normal sex hormone levels [22]. Pollen has also been reported to have beneficial effects on the cardiovascular, respiratory, and nervous systems, treatment of wounds and inflammation [23], and enhancing the effects of pharmacological cancer therapies [24]. In cosmetics, bee pollen can effectively enhance protective mechanisms against skin ageing, skin dryness, ultraviolet B radiation, oxidative damage, inflammation, and melanogenesis [25].

Propolis is a complex resinous mixture used by bees mainly to build their hives. It comprises resin, wax, pollen, essential oils, and organic compounds such as amino acids, phenolic compounds, flavonoids, alcohols, terpenes, aromatic esters, aldehydes, alcohols, or vitamins [1]. Propolis has anticancer, antiviral, anti-fungal, antioxidant, and antihistaminic effects [26]. It accelerates wound healing, relieves gum disease, and is used in treating recurrent vulvovaginal candidiasis and gastric ulcers and as an adjunct in breast cancer therapy [27]. Beneficial effects of propolis on immunity, nervous and cardiovascular functioning [28,29], and the respiratory system [30] have also been reported. Propolis polyphenols support the intestinal microflora, inhibiting the growth of pathogenic bacteria [31]. It also helps to maintain a healthy body weight [32]. The use of propolis in cosmetics is related to its beneficial bacteriostatic and regenerative effects on the skin, including the stimulation of collagen production [33].

Royal jelly is the secretion of worker bees, providing food for the queen bee and the larvae during the first days of life [34]. There are over 200 chemicals in royal jelly, including essential amino acids, enzymes, peptides, lipids, carbohydrates, bioelements, organic acids, nucleic acids, hormones, vitamins, enzymes, and nucleotides. Royal jelly has antimicrobial [35], antioxidant [36], anti-inflammatory [37], anticancer [38], neuroprotective [39], and hypocholesterolemic [40] effects. This product positively affects menstrual syndrome and improves the quality of life of postmenopausal women [41]. Regular consumption of royal jelly affects the treatment of male fertility disorders [42]. Royal jelly is also beneficial for improving metabolism and lipid profiles in diabetes [43]. In the dermatological and cosmetic aspects, royal jelly shortens the wound healing process and stimulates collagen production [27]. It also has a beneficial effect on skin, hair, and nails by supporting the production of collagen fibres and regulating the secretory functions of the sebaceous glands [44].

Bee wax is a natural lipid produced by the wax glands of worker bees. This product contains over 300 substances, including fatty acid esters, hydrocarbons, fatty acids, alcohols, flavonoids, carotenoids, and proteins [45]. Beeswax is characterised by its antiseptic, regenerative, and strengthening properties [21]. In medicine, it is used to treat inflammation of joints, muscles, and nerves. Due to its antiseptic and moisturising properties, beeswax shortens the wound healing period and accelerates epidermal regeneration. In cosmetics, beeswax is used to make creams, body lotions, and ointments. Its addition firms smooth and improve the skin’s condition [21]. As a substance that provides elasticity, plasticity, and increased skin adhesion, beeswax is the base for lipsticks, sticks, and creams [46].

Research to date on bee products focuses on understanding their composition, properties, and potential therapeutic effects [47]. Research papers addressing consumer use of bee products are mainly concerned with honey and focus on the frequency of consumption, reasons for consumption, places of purchase, determinants of choice, and modes of consumption [48,49,50,51,52].

To the best of the authors’ knowledge, there is a lack of research on the motives and ways of the use of honey by consumers for cosmetic and medicinal purposes. There is also a lack of up-to-date research on consumer use of bee products other than honey. In addition, among the determinants of bee product use considered in the analyses to date, self-assessment of nutritional knowledge and health status have not been taken into account. Both of these factors, as proven in studies [53,54,55], can significantly modify dietary preferences and behavior. Several studies have also found that health-promoting properties are an important motivation for the consumption of honey and other bee products [56,57,58].

To complement mentioned above research gaps, a study was undertaken to determine the prevalence of consumers’ use of honey (for culinary, cosmetic, and medicinal purposes) and other bee products, with particular attention to the impact of nutritional knowledge and health status on the variation of these behaviours.

In the context of the research idea thus defined, the following specific objectives were formulated:

- Determination of the frequency of use of honey for culinary, medicinal, and cosmetic purposes;
- Determination of the ways in which honey is used for culinary and cosmetic purposes and the health reasons conditioning its therapeutic use;
- Determination of the prevalence of the use of bee products other than honey for culinary, medicinal, and cosmetic purposes;
- Segmentation of consumers who use honey and other bee products with different frequencies;
- Assessment of the relationship between the level of nutritional knowledge of consumers and their use of honey and other bee products;
- Assessment of the relationship between consumers’ health status and their use of honey and other bee products.

## 2. Materials and Methods

### 2.1. Study Design and Participants

The paper is based on a questionnaire research study conducted in 2022 with the CAWI (Computer-Assisted Web Interview) method. The survey was carried out in full observance of the national and international regulations compliant with the Declaration of Helsinki (2000). The personal information and data of the participants were anonymous, according to the General Data Protection Regulation of the European Parliament (GDPR 679/2016).

The ethical aspects followed throughout the study ensured the participants’ continued safety and the integrity of the accumulated data. A brief description of the study and its aim and the declaration of anonymity and confidentiality were given to the participants before the start of the questionnaire. Respondents did not provide their names or contact information (including IP addresses) and could finish the survey at any stage. The answers were saved only when participants clicked the “submit” button after filling in the questionnaire.

This study was conducted using the Google Forms web survey platform. The sample selection was non-random. The link to the online survey was shared through social media, such as Facebook, Instagram, and WhatsApp, and by personal contacts of the research group members. A snowball effect was targeted in order to attract as many respondents as possible -the participants were asked to share the study link to increase the number of persons who received the invitation to the study and thus increase study participants.

### 2.2. Questionnaire

The questionnaire consisted of three sections. The first concerned the characteristics of respondents by gender, age, education, income, place of residence, and the number of members in a household.

The second part of the questionnaire included questions on the frequency of using honey for culinary, cosmetic, and medicinal purposes (questions on a scale: 0—not at all, 1—several times a year or less, 2—once a month on average, 3—once a week on average, 4—several times a week, 5—daily or almost daily), ways of using honey for culinary, cosmetic and medicinal purposes (questions on a scale: 0—not at all, 1—several times a year or less, 2—once a month on average, 3—once a week on average, 4—several times a week, 5—daily or almost daily), the relevance of selected reasons of bee products usage (1—unimportant, 2—rather not important, 3—moderately important, 4—somewhat important, 5—very important), sources of information about bee products (question in nominal multiple-choice scale).

The questions in the third part of the questionnaire concerned health status and the respondents’ level of nutritional knowledge. Respondents’ health status was assessed using the WHO five-point self-assessment scale of health status (1—very bad, 2—bad, 3—average, 4—good, 5—very good) [59]. An analogous scale was used to measure the self-assessment of respondents’ nutritional knowledge.

### 2.3. Statistical Analysis

Descriptive statistics were used to calculate frequencies and averages. The cluster analysis was carried out based on the frequency of using honey for culinary, cosmetic, and medical purposes. The hierarchical method was used to isolate the clusters. The number of clusters was chosen on the grounds of the dendrogram. The obtained segments were statistically evaluated using CCC (Cubic Clustering Criteria) statistics and pseudo-F statistics. The results of both statistics confirmed that the obtained segments are well separated. The distinguished segments’ profiling process was ensured, considering demographic and economic characteristics such as gender, age, education, income, place of residence and the number of members in a household, results of self-assessment of nutritional knowledge and health status, as well as such issues as usage of other than honeybee products, reasons for bee products usage and source of information about bee products. The comparison of the features mentioned above in individual segments was ensured using the Chi2 independence test at the *p* < 0.05 level of statistical significance.

The relationship between the level of nutritional knowledge and health status of consumers and their behaviour towards bee products was verified using a logistic regression model describing the relationship between the level of nutritional knowledge (1—very high and high nutrition knowledge score, 0—medium, low and very low nutrition knowledge score) and health status (1—very high and high self-assessment of health status, 0—medium and low self-assessment of health status) and the variables describing consumer behaviour towards bee products included in the study.

All calculations were made using the SAS 9.4 statistics package.

### 2.4. Characteristics of Respondents

The research sample consisted of 487 people, of which 57% were women and 43% were men. The age structure of the studied group was: 18–30 years old (43.1%), 31–45 (26.7%), 46–60 years old (18%), and older (12.2%). In terms of education, the largest group was people with higher education (60.6%), 35.7% of the respondents had completed secondary education, and 3.7% had completed vocational or primary education. The analysis of the economic situation of the respondents showed that 33% had a monthly income less than PLN 2500, 40.8% in the range of PLN 2500–5000, and 26.3% declared income more than PLN 5500 per person per month. Regarding the place of residence, 23% of respondents lived in rural areas, 17.5% in small towns, 16.4 in medium-sized cities, and 43.1% in cities with populations over 250,000. In total, 12.2% of respondents formed one-person households, 31.8% were members of two-person households, 25.1% of three-person households, 19.6% of four-person households, and 13.3% of five-person households or more. Level of nutritional knowledge was rated very good by 14.8% of those surveyed, good by 45.6%, and sufficient by 36.8%, while only 3.2% of respondents described it as insufficient. The vast majority of those surveyed (74%) rated their health status as very good or good, while 26% of respondents considered their health status average, bad, and very bad (Table 1).

## 3. Results

### 3.1. Bee Products Usage for Consumption, Cosmetic and Medical Purposes

**Figure 1:**
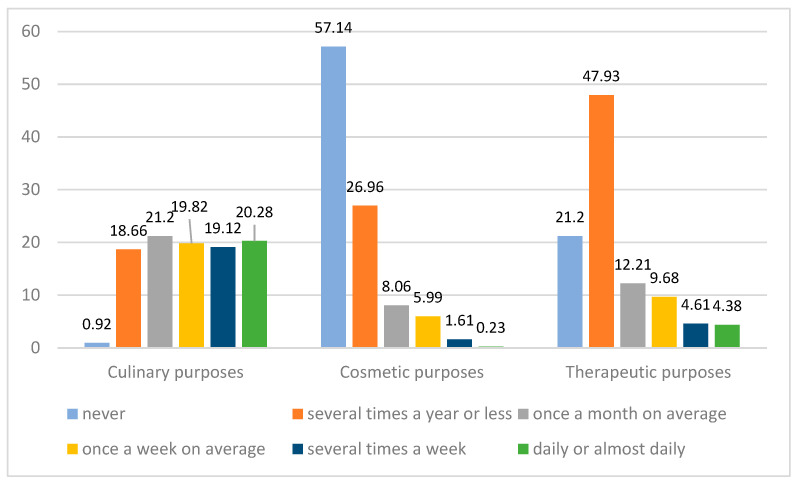
Frequency of honey usage for culinary, cosmetic, and medical purposes (n = 487, data in %).

The survey results indicate that honey is most often used for culinary purposes—less than 1% of the respondents indicated that they do not use honey for this purpose, with individual frequency ranges indicated by about 20% of the respondents each. The use of honey for cosmetic purposes was declared by 42.9% of respondents, with 27% using honey for this purpose several times a year or less frequently, 8.1% once a month on average, 6% several times a month, and only 2% using it more frequently. For medicinal purposes, 79% of respondents use honey, with 48% using it several times a year or less frequently, 12.2% using it once a month on average, 9.7% using it several times a month, and just under 10% using it more frequently (Figure 1). The differences found were statistically significant. A table with detailed data is provided as Supplementary Material Table S1.

**Figure 2:**
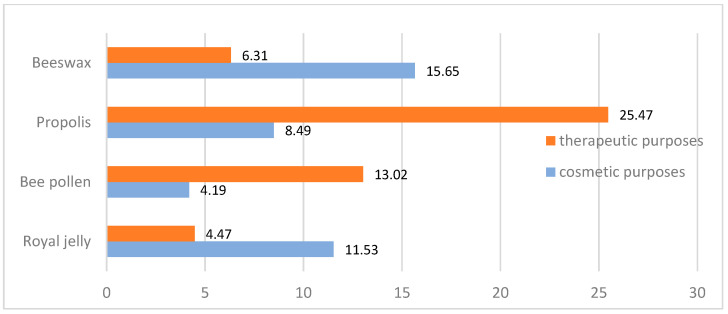
Use of wax, propolis, royal jelly, and bee pollen for cosmetic and medicinal purposes (n = 487, data in %).

Other bee products are used by respondents much less frequently than honey, with none being used for culinary purposes. For cosmetic purposes, beeswax (15.7%) and royal jelly (11.5%) are most commonly used, while propolis (8.5%) and bee pollen (4.2%) are used less frequently. For medicinal purposes, the largest percentage of respondents reported using propolis (25.5%), significantly fewer respondents used bee pollen (13%), and the least used beeswax and royal jelly (6.3% and 4.55, respectively) (Figure 2). The differences found were statistically significant. A table with detailed data is provided as Supplementary Material Table S2.

### 3.2. Ways of Honey Usage for Culinary, Cosmetic, and Medical Purposes

**Figure 3:**
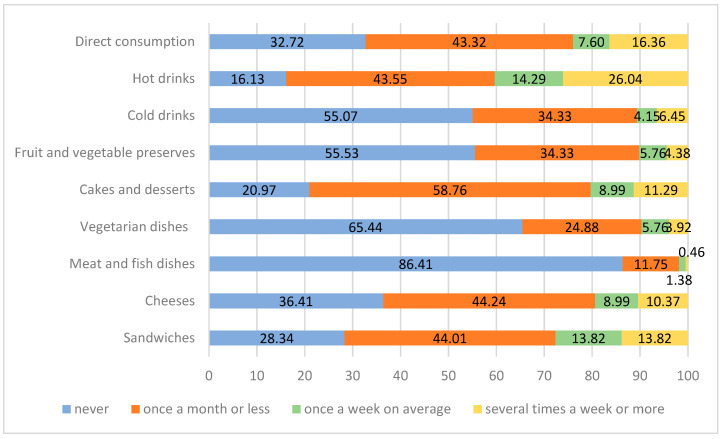
Ways of culinary use of honey (n = 483, data in %).

Analysis of the ways of using honey for culinary purposes showed that respondents declaring such use of honey (n = 483) most commonly apply it to hot drinks (26%) and for direct consumption (16.4%). A smaller proportion of respondents (10–14%) used honey once a week or more often for sandwiches, cheese, cakes, desserts, and cold drinks. Respondents are least likely to use honey for fish or meat dishes, with 86.4% declaring that they do not use honey in this way (Figure 3).

**Figure 4:**
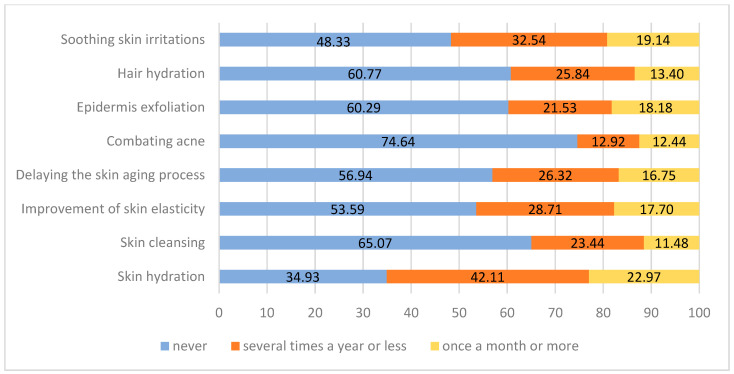
Purposes of cosmetic use of honey (n = 209, data in %).

For cosmetic purposes, honey users (n = 209) are most likely to use it to moisturise the skin (23% at least once a month), to soothe irritation (19.1%), to cleanse the skin (18.25), to improve skin elasticity (17.7%), and to stop the ageing process (16.8%). Honey is least frequently used for anti-acne therapy and skin cleansing, with 74.6% and 65.1% of respondents declaring that they never use honey for these purposes (Figure 4).

**Figure 5:**
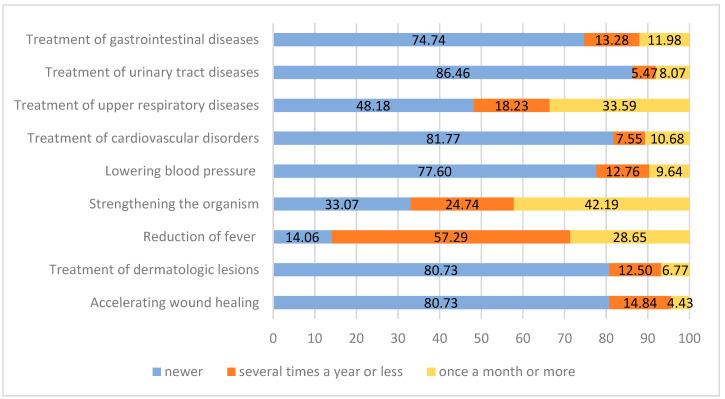
Purposes of medicinal use of honey (n = 384, data in %).

The use of honey for medical purposes was declared by 384 respondents. The most frequently indicated reason was to strengthen the body (42.2% of respondents indicated a frequency of once a month or more often, 24.7% several times a year or less often), to treat upper respiratory tract ailments (33.6% and 18.2%, respectively) and to lower body temperature (28.7% and 57.3%). Honey is used much less frequently to treat gastrointestinal ailments (12% and 13.3%), for lowering blood pressure (9.6% and 12.8%), for cardiovascular diseases treatment (10.7% and 7.6%), and occasionally for conditions such as urinary tract diseases, skin diseases, and wound treatment (Figure 5).

### 3.3. Consumer Segmentation

Segmentation of respondents taking into account the frequency of their use of honey for culinary, cosmetic, and medicinal purposes allowed distinguishing three groups of consumers. The first of these, accounting for nearly 28% of the surveyed population, comprised respondents using honey almost exclusively for culinary purposes. This group was called Honey eaters (HE). The second group, which included 44.6% of the respondents, consisted of people who rarely use honey in all analysed aspects—it was named Honey rarely users (HRU). The third group, comprising 16.6% of respondents, consisted of people who use honey most frequently for cooking, cosmetic purposes, and the prevention and treatment of various diseases. This group was referred to as Honey users (HU) (Table 2).

#### 3.3.1. Demographic and Economic Profile of the Segments

Analysis of the demographic and economic profile of the identified clusters showed that the HE group is characterised by a higher proportion of men, older people, and a higher proportion of respondents with higher income than in the general population. The HRU segment is characterised by a higher share of women, the youngest people and those aged 46–60, and respondents with the lowest income. In contrast, the HU group has almost twice the proportion of women compared to men, the highest share of respondents from the oldest age group and those at a middle-income level (Table 3). No statistically significant differences were found between the separate segments concerning education, place of residence, and number of persons in the household.

#### 3.3.2. Use of Other Than Honeybee Products by Distinguished Segments

An analysis of the interest in the use of other than honeybee products by representatives of the different segments showed that the members of the HE group least frequently use bee products other than honey for cosmetic and medicinal purposes. Representatives of the second separate group (HRU) use bee products most frequently for cosmetic purposes, while relatively rarely for medicinal reasons. In contrast, people in the HU cluster commonly use bee products for medicinal purposes (Table 4).

#### 3.3.3. Reasons for the Use of Bee Products by Distinguished Segments

Of the analysed reasons for the use of bee products, only in the case of habit and suggestions from doctors and nutritionists were statistically significant differences noted between the separate clusters. The former reason motivates the behaviour of the HE and HU segments more, while the latter reason was most significant for the representatives of the HU cluster (Table 5).

#### 3.3.4. Sources of Information on Bee Products Used by the Identified Segments

Of the potential sources of information about bee products included in the analysis, the representatives of the HE cluster relatively more often indicated family and friends as well as books and magazines. The HRU segment was distinguished by the greatest information importance of family and friends, while other sources of information were more important for the representatives of the HU cluster, especially advice obtained from doctors and nutritionists (Table 6).

#### 3.3.5. Self-Assessment of Nutritional Knowledge and Health Status in Distinguished Segments

The respondents representing the HE segment rated their level of nutritional knowledge as the lowest. Among the representatives of the HRU cluster, there were relatively more indications of “average” answers, while the highest percentage of indications of “good” and “very good” answers to the share of the population were recorded in the HU group.

For self-assessed health status, the highest percentages of indications of ‘very bad and bad’ and ‘average’ responses were found among HRUs, while representatives of the HE and HU segments are more likely to perceive their health status as very good (Table 7).

### 3.4. Influence of Nutritional Knowledge and Health Status on the Use of Bee Products

The relationship between the level of nutritional knowledge and consumer behaviour concerning bee products was analysed using logistic regression models. The results obtained allow concluding that a higher level of nutritional knowledge is declared by respondents using honey to treat gastrointestinal ailments (OR: 1.407; 95% Cl: 1.03–1.93) and respondents using propolis for medicinal purposes (OR: 1.682; 95% Cl: 1.29–3.01), while a lower level of nutritional knowledge is associated with the use of bee products as a result of deteriorating health (OR: 0.456; 95% Cl: 0.27–0.76), as well as being influenced by suggestions from family and friends (OR: 0.705; 95% Cl: 0.44–0.91) and doctors and dieticians (OR: 0.419; 95% Cl: 0.23–0.77) (Table 8).

Analysing the association between health status and consumer attitudes towards bee products, it was found that a positive predictor of good health status is the frequent use of propolis for medicinal purposes (OR: 2.385; 95% Cl: 1.42–4.01) and the use of honey to treat gastrointestinal ailments (OR: 1.744; 95% Cl: 1.25–2.44), while a factor lowering the odds of a positive self-assessment of health status is the frequent use of bee products as a result of deteriorating health (OR: 0.488; 95% Cl: 0.3–0.79) (Table 9).

## 4. Discussion

The research aimed to determine the extent and conditions of using honey and other bee products. The first of the detailed goals was to explore consumer behaviour with regard to bee products in terms of their frequency of use for culinary, cosmetic, and medicinal purposes. The analyses conducted showed that honey is most often used for culinary purposes—99% of the respondents used it as such, with varying frequency. The use of honey for cosmetic purposes was declared by 43% of respondents, while honey was used for medicinal purposes by 79% of respondents. The prevalence of honey consumption is also confirmed by other studies conducted in Poland [60,61] and other countries [58,62,63]. The popularity of the therapeutic use of honey is indirectly confirmed by the results of studies carried out in the Czech Republic [56]. Malaysia [57] and Saudi Arabia [64] indicate that the beneficial health effects of honey are an important reason for its consumption.

One of the research intentions was also to determine the ways of using honey for culinary and cosmetic purposes and the health reasons for its therapeutic use. The data obtained show that, in the culinary aspect, respondents most often use honey for hot drinks and direct consumption, as well as for sandwiches, cheese, cakes, desserts, and cold drinks. Most of the indicated uses of honey are related to its use as a sugar substitute, which was also found in the study of Guziy et al. [65] undertaken in Slovakia and Russia. On the other hand, studies carried out in Australia [50], and Romania [66] noted that consumers often use honey as a sandwich spread, as in Poland. When using honey for cosmetic purposes, respondents apply it most often to moisturise, soothe irritation, and cleanse the skin, as well as to improve its elasticity and slow down the ageing process. The validity of this use of honey is confirmed by the results of the studies [19,20]. In the medical context, respondents’ main reasons for using honey were to strengthen the body and treat upper respiratory conditions and lower body temperature. The therapeutic uses of honey indicated by the respondents have scientific justification, as it has scientifically proven immunomodulatory and antiseptic properties [13].

Another detailed research objective was to determine the prevalence of the use of bee products other than honey. The results showed that they were used much less frequently than honey, and none were used for culinary purposes. For cosmetic purposes, beeswax and royal jelly were the most widely used, while propolis and bee pollen were the most extensively used for medicinal purposes. Additionally, a study in Slovakia [67] noted that propolis and bee pollen are the most commonly used bee products besides honey and mead.

Segmentation of respondents taking into account the frequency of honey use for culinary, cosmetic, and medicinal purposes allowed three groups of consumers to be distinguished. The first segment (Honey eaters) accounted for 28% of the study population and was characterised by using honey almost exclusively for culinary purposes. It was marked by a higher proportion of men, older people, and respondents with higher incomes. The second segment (Honey rarely users), which included 44.6% of the respondents, consisted of people who rarely used honey in all aspects analysed. This group included slightly more women, the youngest people, those aged 46–60, and respondents with the lowest income. The third segment (Honey users) comprised 16.6% of the respondents most frequently using honey for cooking, cosmetic and therapeutic purposes. Compared to the other groups, there were more women, older people, and respondents with a middle-income level among HU. The demographic-economic characteristics of the segments show the influence of gender, age, and income level on the variation in the frequency of honey use. This is confirmed by studies carried out in Hungary and Romania [68,69] in which the effect of education on honey consumption was also noted, which was not found in the authors’ study. Additionally, the place of residence and the number of people in the household did not differentiate between the identified segments, as was found in studies conducted in Turkey [58] and Saudi Arabia [64].

The fact that the isolated segments differed in terms of their use of bee products other than honey seems interesting—the segment interested mainly in the culinary aspect of honey (HE) used it infrequently, the group rarely using honey (HRU), used it mainly for cosmetic purposes, while those who most often use honey for cooking, cosmetics and therapeutic goals (HU), used other bee products more frequently for medicinal purposes. This may be related to the demographic and economic characteristics of the segments. There were relatively more women and younger people in the HRU group, which is conducive to the interest in natural cosmetic preparations [70,71]. In contrast, a greater proportion among HU of older people, who are more likely to use dietary supplements [72], may explain the more common use of bee products other than honey for medicinal purposes in this group.

Also noteworthy is the fact that the separate segments were differentiated according to the level of nutritional knowledge and health status—the highest ratings in both categories were indicated by representatives of the HU segment and the lowest by HRU. This leads to the conclusion that nutritional knowledge is a motivating factor for using honey and other bee products and that the use of these products results in a higher assessment of health status. The beneficial effect of nutritional knowledge for those on a diet has also been shown by other studies [54,73], and the therapeutic value of honey and other bee products was presented in the introduction of this article.

The association between respondents’ level of nutritional knowledge and self-assessed health status and their use of honey and other bee products was also assessed using logistic regression. It showed that a higher level of nutritional knowledge and better health status were associated with the use of propolis for therapeutic purposes and the use of honey to treat gastrointestinal ailments, whereas a lower score of nutritional knowledge and a poorer self-assessment of health status were correlated with the use of bee products because of deteriorating health status and, in the case of nutritional knowledge, also as a result of the suggestions of doctors and dieticians. The results suggest that the use of bee products other than honey, as well as the therapeutic and broader use of honey than just for the treatment of colds, may be the result of greater nutritional knowledge and have a beneficial effect on perceptions of health status. However, it should be underlined that these are general suggestions that need to be verified in subsequent studies.

## 5. Conclusions

The research provided insight into Polish consumers’ behaviour concerning bee products. It was found that honey was used mainly for culinary purposes (most often for hot drinks and direct consumption), to a lesser extent for medicinal purposes (to strengthen the organism, treat respiratory infections and lower the temperature), and to a minor extent for cosmetic purposes (mainly for moisturising, cleansing, and soothing the epidermis). Other bee products were less commonly used than honey—beeswax and royal jelly were the most commonly used for cosmetic purposes. In contrast, propolis and bee pollen were used for medicinal purposes. Segments distinguished by the frequency of use of honey for particular purposes were differentiated by gender, age, income level, use of other bee products, and motivation to use them. Their differences were also found in terms of the level of nutritional knowledge and self-assessed health status—the highest ratings in both categories were indicated by representatives of the Honey users’ segment. Regression analysis additionally showed that higher levels of nutritional knowledge and better health status were associated with the use of honey to treat gastrointestinal ailments and with the use of propolis for medicinal purposes.

The study results indicate a wide variation in consumer behaviour regarding honey and other bee products and the relationship between the use of these products and the level of nutritional knowledge and health status. The results obtained may be helpful for marketers of bee products in activating consumer buying behaviour and for doctors and nutritionists in finding arguments to encourage their patients to increase bee product consumption.

## Figures and Tables

**Table 1:** Sample characteristics (n = 487, data in %).

**Gender**	
Female	57.14
Male	42.86
**Age**	
18–30 years old	43.09
31–45 years old	26.73
46–60 years old	17.97
Over 60 years old	12.21
**Education**	
Primary, vocational	3.69
Secondary	35.71
Higher	60.60
Per Capita Income PLN/EUR *	
Under 2500 PLN/534.2 EUR	32.95
2501–5000 PLN/534.4–1068.4 EUR	40.78
Above 5000 PLN/1068.4 EUR	26.27
**Place of residence**	
Village	23.04
City up to 50 thousand residents	17.51
City of 50–250 thousand inhabitants	16.36
City of more than 250 thousand inhabitants	43.09
**Number of household members**	
1	12.21
2	31.80
3	25.12
4	19.59
5 and more	13.29
**Nutritional knowledge**	
Very bad and bad	3.23
Average	36.41
Good	45.61
Very good	14.75
**Health self-assessment**	
Very bad and bad	5.76
Average	20.28
Good	54.15
Very good	19.81

* As of 14.12.2022, Polish National Bank exchange rates.

**Table 2:** Results of cluster analysis based on the frequency of honey use for culinary, cosmetic, and medicinal purposes (n = 487, data in %).

Specification Share in the Study Population	HE 27.92	HRU 44.55	HU 16.63	*p*-Value
Frequency of use of honey for culinary purposes	4.01 *^b^	1.78 ^c^	4.48 ^a^	<0.0001
Frequency of use of honey for cosmetic purposes	0.22 ^c^	0.64 ^b^	1.60 ^a^	<0.0001
Frequency of use of honey for medicinal purposes	0.77 ^c^	1.18 ^b^	3.15 ^a^	<0.0001

* on a 5-point scale ranging from n—number of respondents. ^a,b,c^ different superscripts in each line indicate significant differences between identified clusters (ANOVA with posthoc Waller–Duncan K-ratio *t*-test, *p* < 0.05).

**Table 3:** Clusters characteristics by demographic and economic features (n = 487, data in %).

Specification		HE 27.87	HRU 44.55	HU 16.63	*p*-Value
Share in the Study Population					
Gender	Female	22.98	54.04	22.98	<0.0001
	Male	44.47	42.63	12.91	
Age	18–30	27.81	56.15	16.04	0.0089
	31–45	37.93	43.97	18.10	
	46–60	32.05	53.85	14.10	
	Over 60 years	28.30	35.85	35.85	
Income (per capita)	Under PLN 2500	23.78	62.23	13.99	0.0150
	PLN 2501–5000	33.33	43.51	23.16	
	Above PLN 5000	37.72	44.74	17.54	

Chi test of independence^2^. Statistically significant (*p* < 0.05).

**Table 4:** Clusters characteristics by usage other than honeybee products (n = 487, data in %).

Specification Share in the Study Population		HE 27.87	HRU 44.55	HU 16.63	*p*-Value
Use of royal jelly for cosmetic purposes	No	34.57	47.88	17.55	0.0008
	Yes	8.16	69.39	22.45	
Use of bee pollen for cosmetic purposes	No	32.52	48.55	18.93	0.0301
	Yes	5.56	77.77	16.67	
Use of propolis for cosmetic purposes	No	32.47	47.17	20.36	0.0001
	Yes	13.89	83.33	2.78	
Use of bee wax for cosmetic purposes	No	34.35	47.64	18.01	0.0070
	Yes	14.93	61.19	23.88	
Use of royal jelly for medicinal purposes	No	32.51	51.23	16.26	<0.0001
	Yes	10.53	31.58	57.89	
Use of bee pollen for medicinal purposes	No	33.96	48.93	17.11	0.0042
	Yes	14.29	55.35	30.36	
Use of propolis for medicinal purposes	No	30.70	56.33	12.97	<0.0001
	Yes	31.48	32.41	36.11	
Use of bee wax for medicinal purposes	No	31.42	51.37	17.21	0.0013
	Yes	29.63	25.93	44.44	

Chi test of independence^2^. Statistically significant (*p* < 0.05).

**Table 5:** Clusters characteristics by reasons for using bee products (n = 487, data in %).

Specification Share in the Study Population		HE 27.87	HRU 44.55	HU 16.63	*p*-Value
Personal preferences	No	23.4	57.45	19.15	0.1530
	Yes	33.53	47.94	18.53	
Suggestions from family and friends	No	36.50	46.00	17.50	0.0996
	Yes	26.92	53.42	19.66	
Habits	No	27.19	57.46	15.35	0.0045
	Yes	35.92	41.75	22.33	
Deterioration of health	No	31.99	48.45	19.57	0.5184
	Yes	29.46	54.46	16.07	
Suggestions from doctors and nutritionists	No	33.06	49.73	17.21	0.0470
	Yes	22.06	51.47	26.47	
Fashion	No	31.44	50.12	18.44	0.7579
	Yes	27.27	45.45	27.27	

Chi test of independence^2^. Statistically significant (*p* < 0.05).

**Table 6:** Clusters characteristics by sources of information about bee products (n = 487, data in %).

Specification Share in the Study Population		HE 27.87	HRU 44.55	HU 16.63	*p*-Value
Family and friends	No	32.54	42.6	24.85	0.0121
	Yes	30.57	54.72	14.72	
Internet nutrition portals	No	33.19	52.40	14.41	0.0453
	Yes	29.27	47.32	23.41	
Books and magazines	No	31.16	49.66	19.18	0.9250
	Yes	31.69	50.7	17.61	
Vendors	No	34.2	50.49	15.31	0.0104
	Yes	24.41	48.82	26.77	
Social media	No	32.04	51.50	16.47	0.0491
	Yes	29.00	45.00	26.00	
Television and radio	No	31.78	50.65	17.57	0.2452
	Yes	27.66	44.68	27.66	
Doctors and nutritionists	No	32.74	50.64	16.62	0.0030
	Yes	18.6	44.19	37.21	

Chi test of independence^2^. Statistically significant (*p* < 0.05).

**Table 7:** Clusters characteristics by nutritional knowledge and health status (n = 487, data in %).

Specification Share in the Study Population		HE 27.87	HRU 44.55	HU 16.63	*p*-Value
Self-assessment of nutritional knowledge	Very bad and bad	57.14	35.72	7.14	0.0067
	Average	32.91	55.70	11.39	
	Good	29.29	46.47	24.24	
	Very good	28.13	50.00	21.87	
Self-assessment of health status	Very bad and bad	16.00	56.00	28.00	0.0072
	Average	27.68	62.09	10.23	
	Good	29.36	51.92	18.72	
	Very good	41.86	33.72	24.42	

Chi test of independence^2^. Statistically significant (*p* < 0.05).

**Table 8:** Association of nutritional knowledge with bee products-related consumer behaviour (logistic regression results).

Specification	Variable Level	Estimate	Point Estimate	95% Wald Confidence Limits		Pr > ChiSq
Intercept		0.301				0.5635
Use of honey to treat digestive ailments	Yes No (ref.)	0.3416 0	1.407	1.03	1.93	0.0345
Use of propolis for medicinal purposes	Yes	0.5202	1.682	1.29	3.01	0.0379
	No (ref.)	0				.
Deterioration of health as a reason for using bee products	Yes	−0.7856	0.456	0.27	0.76	0.0026
	No (ref.)	0				.
Suggestions from doctors and nutritionists as a reason for using bee products	Yes	−0.8698	0.419	0.23	0.77	0.0050
	No (ref.)	0				

**Table 9:** Association of health status with bee products-related consumer behaviour (logistic regression results).

Specification	Variable Level	Estimate	Point Estimate	95% Wald Confidence Limits		Pr > ChiSq
Intercept		−0.1831				0.698
Use of honey to treat digestive ailments	Yes	0.55590	1.744	1.25	2.44	0.0011
	No (ref.)					
Use of propolis for medicinal purposes	Yes	0.869	2.385	1.42	4.01	0.001
	No (ref.)	0				
Deterioration of health as a reason for using bee products	Yes	−0.7179	0.488	0.30	0.79	0.0035
	No (ref.)	0				.

## Data Availability

The authors confirm that the datasets analysed during the study are available from the corresponding author upon reasonable request.

## Supplementary Materials

Following supporting information can be downloaded at: https://www.mdpi.com/article/10.3390/nu15030737/s1, Table S1: Statistical significance analysis of the variation in the frequency of use of honey for consumption, cosmetic and medicinal purposes (Chi^2^ test); Table S2: Statistical significance analysis of the variation in the use of bee products other than honey for cosmetic and medicinal purposes (Chi^2^ test).
