# Supplementary material for: Determinants of Honey and Other Bee Products Use for Culinary, Cosmetic, and Medical Purposes
Source: Nutrients. 2023 Feb 1;15(3):737. doi: 10.3390/nu15030737 (PMC9919050; doi:10.3390/nu15030737)
Supplement: Supplementary file 1 [file nutrients-15-00737-s001.zip › nutrients-2163036-supplementary.pdf]

Table S1. Statistical significance analysis of the variation in the frequency of use of honey for consumption, cosmetic and medicinal purposes (Chi² test).

	**Consumption purposes**	**Cosmetic purposes**	**Therapeutic purposes**	**Total**	**p**
**never**	4	248	92	344	p<.0001
%col	0,92%	57,14%	21,20%		
%row	1,16%	72,09%	26,74%		
**several times a year or less**	81	117	208	406	
%col	18,66%	26,96%	47,93%		
%row	19,95%	28,82%	51,23%		
**once a month on average**	92	35	53	180	
%col	21,20%	8,06%	12,21%		
%row	51,11%	19,44%	29,44%		
**once a week on average**	86	26	42	154	
%col	19,82%	5,99%	9,68%		
%row	55,84%	16,88%	27,27%		
**several times a week**	83	7	20	110	
%col	19,12%	1,61%	4,61%		
%row	75,45%	6,36%	18,18%		
**daily or almost daily**	88	1	19	108	
%col	20,28%	0,23%	4,38%		
%row	81,48%	0,93%	17,59%		

Table S2. Statistical significance analysis of the variation in the use of bee products other than honey for cosmetic and medicinal purposes (Chi² test).

	**Cosmetic purposes**	**Therapeutic purposes**	Total	
**Royal jelly**	49	19	68	p<.0001
%col	28,82%	9,05%		
%row	72,06%	27,94%		
**Bee pollen**	18	56	74	
%col	10,59%	26,67%		
%row	24,32%	75,68%		
**Propolis**	36	108	144	
%col	21,18%	51,43%		
%row	25,00%	75,00%		
**Beeswax**	67	27	94	
%col	39,41%	12,86%		
%row	71,28%	28,72%		
